# A Hadoop-Based Method to Predict Potential Effective Drug Combination

**DOI:** 10.1155/2014/196858

**Published:** 2014-07-23

**Authors:** Yifan Sun, Yi Xiong, Qian Xu, Dongqing Wei

**Affiliations:** State Key Laboratory of Microbial Metabolism and College of Life Science and Biotechnology, Shanghai Jiao Tong University, Shanghai 200240, China

## Abstract

Combination drugs that impact multiple targets simultaneously are promising candidates for combating complex diseases due to their improved efficacy and reduced side effects. However, exhaustive screening of all possible drug combinations is extremely time-consuming and impractical. Here, we present a novel Hadoop-based approach to predict drug combinations by taking advantage of the MapReduce programming model, which leads to an improvement of scalability of the prediction algorithm. By integrating the gene expression data of multiple drugs, we constructed data preprocessing and the support vector machines and naïve Bayesian classifiers on Hadoop for prediction of drug combinations. The experimental results suggest that our Hadoop-based model achieves much higher efficiency in the big data processing steps with satisfactory performance. We believed that our proposed approach can help accelerate the prediction of potential effective drugs with the increasing of the combination number at an exponential rate in future. The source code and datasets are available upon request.

## 1. Introduction

In the past few years, the novel effective drugs come out slowly although there is a substantial investment into the development of drugs. It is common for the pharmaceutical industry to develop novel drugs targeting a certain target. However, the once dominating paradigm of “mono drug mono target” in drug development is now being challenged by the clinical and pharmaceutical people, since the single drug cannot always be effective for the complex diseases (such as cancer and diabetes), which may involve multiple biological pathways and complex pathological process. Therefore, the drug combination, which consists of multiple drugs (the effective chemical molecules), is now becoming a novel strategy to combat complex diseases [1–3].

It is impractical to screen all possible drug combinations experimentally since there will be an exponential explosion when the number of single drugs increases. Therefore, a great number of computational methods have been recently developed for prediction of drug combinations [4–7]. In general, there are three main kinds of computational approaches to identify effective drug combinations: the method of the first kind is to use the stochastic search technique, which is successfully applied in various applications to solve the large-scale combinatorial optimization problems of highly complex systems, and the fast convergence can be achieved with a small number of iterations to find effective drug combinations [5]; the second type is to build a mathematical model based on the median-effect equation in which the “median” is the unified common link of single entity and multiple entities. The disadvantage of this method is that it is hard to interpret the molecular mechanism that underlies the drug combinations [6]; the third type is based on the systems biology principle, which aims to study the possible effect of the various drug combinations on the molecular networks or pathways which they may be involved in. For example, Zhao et al. [4] integrated the molecular and pharmacological features of drugs to predict new potential drug combinations. Wu et al. [7] assumed that the single drug or the drug combinations affected a subnetwork or pathway in the cellular system. They proposed a molecular interaction network-based method to identify effective drug combinations by evaluating the overall effect of one drug or drug combinations.

Although these existing methods can predict novel drug combinations or provide mechanistic insights into existing ones, they are limited by their efficiency when the size of combination space increases at an exponential growth rate (e.g., the number of drugs increases from pairwise combinations to three-wise combinations). Therefore, it is necessary to develop prediction methods that are scalable to data and computation. The Hadoop MapReduce system [8–10] represents a novel program framework with the potential to greatly accelerate data-intensive application. In the present study, we developed the Hadoop-based method to identify the potential effective drug combinations by integrating the gene expression data under the effect of single drugs, the basic information of drug combination, and human disease pathway information. The classification algorithms were then constructed based on the typical perceptron learning algorithm and generative learning algorithm: support vector machine (SVM) and naïve Bayesian for prediction of novel effective drug combinations. The preliminary results indicated that our Hadoop-based implementation of these classification algorithms achieved higher efficiency than the traditional implementation of the algorithms on the dataset with a small number of samples due to insufficient number of effective drug combinations validated. We believe that the proposed Hadoop-based approach will be useful on the larger dataset when the number of drug combinations greatly increases in future.

## 2. Methods

### 2.1. Datasets

All the basic information about single drugs and effective drug combinations was extracted from the Drug Combination Database (DCDB) (http://www.cls.zju.edu.cn/dcdb/) [11]. In total, our data set contains 76 pairwise drug combinations involving 103 single drugs, which have well annotated gene expression information (more details explained in the next section). The 76 drug combinations were assigned as the positive samples in the classification models, while the noneffective pairs (called the negative set) were generated by randomly pairing drugs that appeared in the set of the 103 single drugs. The negative set meets the two requirements: (i) the noneffective pairs cannot exist in the set of 76 effective pairs, and (ii) the number of noneffective (negative) pairs is equal to that of effective (positive) pairs.

### 2.2. Feature Construction

In order to encode the drug combinations, we focus on the possible effect of different drug combinations on the pathways that they may be involved in. The gene expression profiles of the 1309 small-molecule drugs or compounds were downloaded from the Broad Institute Connectivity Map Build02 (http://www.broadinstitute.org/cmap/) [12], and the size of total data is up to 45 GB. We kept the genes which have microarray experiments with at least 3 replicates. The raw expression profiles were processed by using MAS5 algorithm supplied by Affymetrix, which is much faster than RMA (robust multichip average) running on our limited computing capability [13, 14]. The annotated gene set in each human disease pathway was sourced from the Molecular Signatures Database (MSigDB, http://www.broadinstitute.org/gsea/msigdb/) [15]. We finally got 186 gene sets which are related to the human disease pathways.

For the fact that we can only directly obtain the gene expression data of single drugs, we should first represent the feature of pairwise (or multiple) drug combinations. In this study, we applied two different strategies to define the combination feature described as below.

(1) This first kind of representation is a direct way to define the combination feature as a linear function of single drugs. For a drug *D*_*i*_ in the drug combination (*D*_1_, *D*_2_), the expression data of gene *G*_*i*_ is denoted as *P*_*i*_ if it is not affected by drug *D*_*i*_, and denoted as *C*_*i*_, if it is affected by drug *D*_*i*_. Thus, the combination effect of the pairwise drug combination of *D*_1_ and *D*_2_ on *G*_*i*_ is defined as

(1) $$\begin{array}{c} D_{1,2\;|\;G}=\left(\frac{P_{1\;|\;G}}{C_{1\;|\;G}}-1\right)+\left(\frac{P_{2\;|\;G}}{C_{2\;|\;G}}-1\right). \end{array}$$

Obviously, this is a simple way to get the combination feature of any pairwise drug combination. However, the representation cannot convey the intricacy of drug combinations due to the complexity of human disease mechanism.

(2) Instead, we try another way to find the frequent feature pattern of effective drug combinations and take them as the feature of potential effective drug combinations. Here, we assume that a pathway is affected if there exist genes in this pathway whose expression level is significantly changed under the effect of a single drug. We first performed the Student's* t*-test for each single drug to get the significantly changed gene set and then mapped them into 186 human disease pathways. This method is finally compared with Zhao et al.'s definition [4], which directly maps the target of the drug into human disease pathway. Finally, we calculated the frequency score of all pairwise drug combinations. The frequency score is defined as below:

(2) $$\begin{array}{c} S_{i,j}=\frac{N_{i,j}\left(EC\right)}{N_{i,j}\left(RC\right)}, \end{array}$$

where the denominator shows the number of patterns that emerged in effective pairwise drug combinations and the numerator presents the background frequent patterns in randomly distributed pairwise drug combinations.

### 2.3. Feature Selection

The feature construction method brings high dimensional feature space on a dataset with small size of samples. To avoid the overfitting, we applied several feature selection methods on our dataset. For the first type of feature construction method mentioned above, we performed the minimum-redundancy-maximum-relevance (mRMR) [16] to select the most important feature for model building, whereas, for the second one, we only need to set a fixed threshold to take the most frequent emerging pattern as the features. In this study, we chose the number of features as one-fourth of the total sample number.

### 2.4. Model Construction

In the model building step, we employed two popular machine learning algorithms, support vector machine, and naïve Bayesian to train a classifier for predicting effective drug combinations. In the SVM algorithm, the selection of kernel function and related parameters will have a great effect on the performance of the trained classifier. In the training stage, we compared four types of kernel functions: linear kernel, polynomial kernel, Gaussian kernel, and tangent kernel. The SVM classifiers were implemented by using LibSVM package [17]. There are two important parameters when training SVM classifiers, cost factor* c *for outlier samples and gamma *g* in kernel functions. There is no smart algorithm to select the best parameters in the training stage, and we searched the optimal parameters using grid search. The search range of the parameters (*c* and *g*) is from 0.03125 to 32, with the step as 0.00001. The second type of classification method we used here is the naïve Bayesian algorithm, which can be suitable to be parallelized. In the later section, we will introduce how to implement the MapReduce version of the naïve Bayesian algorithm on the Hadoop platform.

### 2.5. Scalable Implementation of the Whole Mining Process

#### 2.5.1. Building the Big Data Platform

For scalable implementation of our mining process, we used the machine virtualization to build the Hadoop cluster. The master virtual machines included 4 Intel core i3 processor cores and 4 GB RAM and the two slave virtual machines with 2 Intel core i3 processor cores and 2 GB RAM. The software environment includes Hadoop-1.2.1, Hive-0.11.0, and RHadoop (an integration of R and Hadoop).

After building the scalable Hadoop cluster, we exploited the Hadoop distributed file system to store the raw data and used hive as data ETL tools for relational database and program to process the local files.

#### 2.5.2. Scalable Feature Construction

The feature construction stage can be regarded as a series of independent similar processes on different samples and features. In the Hadoop, we implemented a chain mapper to parallelize the processes, including the gene expression preprocessing and the construction of the proposed drug combination features.

#### 2.5.3. Scalable Model Building

For the SVM algorithm, it is difficult to implement the parallel version. Here, we only parallelized the grid search of the optimal parameters, which are time-consuming in the sequential implementation.

For the naïve Bayesian algorithm, the implementation of the scalable version using MapReduce is mainly composed of three steps (shown in Algorithm 1): the calculation of the prior probability for each class, the conditional probability for each feature under each class, and the conditional probability for each class under each feature.

### 2.6. Model Validation and Evaluation

A tenfold cross-validation and leave-one-out cross-validation test were used to evaluate the classification performance. To assess the performance of the classification models, we used the accuracy (ACC), sensitivity (SN, also called recall), specificity (SP), and* F*-measure (*F*_1_). These measures can be calculated by the numbers of true positives (TP), false positives (FP), true negatives (TN), and false negatives (FN) for each classifier [18–21]. These performance measures are defined as below:

(3) $$\begin{array}{c} \text{ACC}=\frac{\text{TP}+\text{TN}}{\text{TP}+\text{FP}+\text{TN}+\text{FN}},\\ \text{SN}=\frac{\text{TP}}{\text{TP}+\text{FN}},\\ \text{SP}=\frac{\text{TN}}{\text{TN}+\text{FP}},\\ F_{1}=\frac{2\times \text{TP}}{\text{FP}+\text{FN}+2\times \text{TP}}. \end{array}$$

## 3. Results

### 3.1. Optimization of the Prediction Model

The performance of the prediction model using SVM algorithm is determined by the representation of the features, the type of the kernel function, and parameters. Here, the tenfold cross-validation test was conducted to evaluate the model performance. We employed three ways of feature representation, including the linear addition, Zhao's frequent pattern [4], and our frequent pattern. We further used four different types of kernel functions, which are linear, polynomial, Gaussian, and Tanh functions. As shown in Table 1, our proposed frequent pattern performed much better than the other two patterns, regardless of the types of the kernel functions. The result in Table 1 also suggests that the Gaussian function achieved higher accuracy than the other types of the kernel functions.

### 3.2. Independent Test

In this section, we evaluated the prediction performance using our proposed frequent pattern and the Gaussian function on the independent test, which is mimicking a true prediction since the model trained on one dataset is used to test on an unseen dataset. We randomly split the whole set of the 76 drug combinations into two datasets (a training set and a testing set). The ratio is about 4 : 1 between the number of the samples of the training set and that of the testing set. The split of the dataset and the independent test is repeated for 10 times. The performance of the 10 runs and their average is presented in Table 2. As shown in Table 2, the model trained by using our proposed frequent pattern performed as well on the independent test, suggesting that our model can predict the unseen data equally well.

### 3.3. Classification by the One-Class SVM Classifier

In the task of the two-class classification, the assignment of the negative samples (noneffective drug combinations) is not perfect since the unknown pairwise drug combination (we now consider it as noneffective drug combination) may be proved to be an effective drug combination in future. To avoid this problem, we constructed the one-class SVM classifier trained on the dataset with only effective drug combinations. We made use of leave-one-out cross-validation to assess the accuracy of one-class SVM classifiers using different types of kernel functions. As shown in Table 3, without the bias of negative samples, the accuracy of SVM classifiers has significantly increased for polynomial, Gaussian, and hyperbolic tangent kernel, while the linear classifier remains at a lower performance. We have also conducted a test on some nonpositive samples, namely, drug combination that has not yet been approved, and also randomly repeated for 10 times, each testing set containing 76 negative samples. The average result of these 10 repeat experiments suggests that 67.1% of the unknown pairwise samples were predicted as noneffective drug combinations, which is consistent with the fact that there exists a low possibility of the effective drug combinations in the large number of randomly chosen pairs of drugs.

### 3.4. Extension to a Scalable Mining Process

In this section, we constructed a scalable version of the mining tool for identifying the effective drug combinations and compared its efficiency to that of the sequential implementation by the traditional way. The preprocessing steps (including microarray processing, single drug, and drug combination feature construction) were parallelized by a chain of mappers. The naïve Bayesian algorithm is implemented by a series of MapReduce jobs.

The detailed comparison results of our scalable version and the sequential version in efficiency are listed in Table 4. It is clearly shown in Table 4 that the scalable version achieved higher efficiency in some big data processing steps such as microarray processing, feature construction, and SVM grid search. For naïve Bayesian, the scalable algorithm did not have the advantage against sequential naïve Bayesian, since our final dataset for model construction and evaluation was quite small. However, we believed that the prediction of drug combinations will benefit from our proposed scalable version with the increasing size of the search space of possible drug combinations in future.

## 4. Conclusions

In this study, we proposed a novel Hadoop-based approach to predict drug combinations by implementing the support vector machine and naïve Bayesian classifiers using the MapReduce programming model, which can advance the improvement of scalability of the prediction algorithm. We believe that our proposed model can be potentially useful when more than two drugs (the increasing availability of the number of the drug combination) are combined for combating the complex diseases in the long run.

## Figures and Tables

**Algorithm 1:**
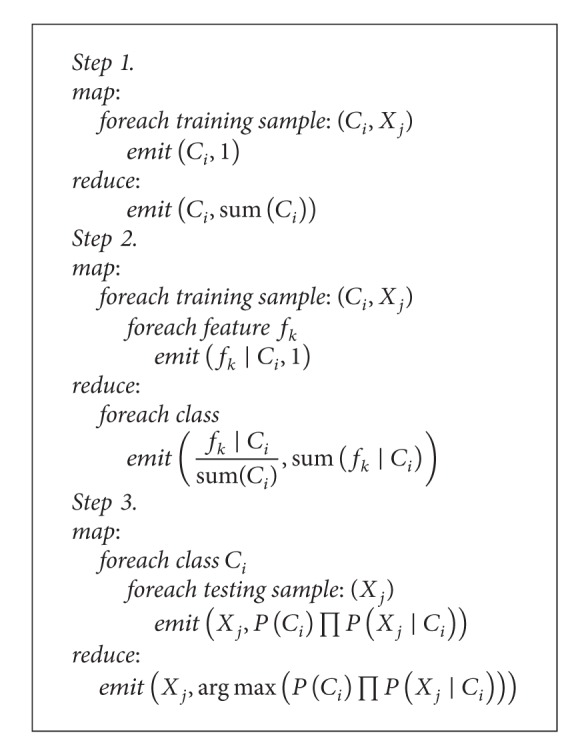
The workflow of the scalable version of the Naïve Bayesian algorithm implemented by MapReduce.

**Table 1:** Comparison of the accuracy of the prediction models based on SVM using various feature representation and kernel functions.

	Linear	Polynomial	Gaussian	Tanh
Linear addition pattern	47.7%	47.7%	47.7%	53.0%
Zhao's frequent pattern [4]	50.0%	55.1%	57.4%	56.2%
Our frequent pattern	62.2%	64.6%	69.1%	65.4%

**Table 2:** The performance of the independent test using our definition of frequent pattern and Gaussian kernel.

Run	ACC	SN	SP	*F*_1_
1	67.7%	70.6%	64.3%	0.706
2	65.0%	54.5%	77.8%	0.632
3	60.9%	44.4%	71.4%	0.471
4	64.0%	66.7%	60.0%	0.690
5	68.2%	61.5%	77.8%	0.696
6	65.5%	41.7%	82.4%	0.500
7	77.8%	64.3%	92.3%	0.750
8	72.2%	76.9%	60.0%	0.800
9	72.0%	66.7%	80.0%	0.741
10	70.4%	66.7%	75.0%	0.714

Average	68.4%	61.4%	74.1%	0.670

**Table 3:** The performance of the one-class SVM classifiers using different kernel functions.

	Linear	Polynomial	Gaussian	Tanh
ACC	46.1%	81.2%	88.2%	80.3%

**Table 4:** Comparison of the average efficiency between the scalable and sequential version.

Mining steps	Scalable version	Sequential version
Microarray processing	2 h 3 min	6 h 18 m
Feature construction	8 min 34 s	18 min 3 s
Naive Bayesian	15 s	3 s
SVM grid search	27 min 6 s	1 h 11 min
